# Reducing the Schizophrenia Stigma: A New Approach Based on Augmented Reality

**DOI:** 10.1155/2017/2721846

**Published:** 2017-11-29

**Authors:** Rafael D. de C. Silva, Saulo G. C. Albuquerque, Artur de V. Muniz, Pedro P. Rebouças Filho, Sidarta Ribeiro, Plácido Rogerio Pinheiro, Victor Hugo C. Albuquerque

**Affiliations:** ^1^Programa de Pós-Graduação em Informática Aplicada, Laboratório de Bioinformática, Universidade de Fortaleza, Fortaleza, CE, Brazil; ^2^Hospital de Saúde Mental de Messejana, Fortaleza, CE, Brazil; ^3^Internato em Saúde Coletiva e Mental, Universidade de Fortaleza, Fortaleza, CE, Brazil; ^4^Laboratório de Processamento de Imagens e Simulação Computacional, Instituto Federal de Educação, Ciência e Tecnologia do Ceará, Maracanaú, CE, Brazil; ^5^Instituto do Cérebro, Universidade Federal do Rio Grande do Norte, Natal, RN, Brazil

## Abstract

Schizophrenia is a chronic mental disease that usually manifests psychotic symptoms and affects an individual's functionality. The stigma related to this disease is a serious obstacle for an adequate approach to its treatment. Stigma can, for example, delay the start of treatment, and it creates difficulties in interpersonal and professional relationships. This work proposes a new tool based on augmented reality to reduce the stigma related to schizophrenia. The tool is capable of simulating the psychotic symptoms typical of schizophrenia and simulates sense perception changes in order to create an immersive experience capable of generating pathological experiences of a patient with schizophrenia. The integration into the proposed environment occurs through immersion glasses and an embedded camera. Audio and visual effects can also be applied in real time. To validate the proposed environment, medical students experienced the virtual environment and then answered three questionnaires to assess (i) stigmas related to schizophrenia, (ii) the efficiency and effectiveness of the tool, and, finally (iii) stigma after simulation. The analysis of the questionnaires showed that the proposed model is a robust tool and quite realistic and, thus, very promising in reducing stigma associated with schizophrenia by instilling in the observer a greater comprehension of any person during an schizophrenic outbreak, whether a patient or a family member.

## 1. Introduction

The World Health Organization (WHO) describes schizophrenia as one of the most serious and challenging psychiatric diseases nowadays, which still demands strong efforts in order to acquire a more solid knowledge on the pathology. It is a complex disease categorized by distortions of thought, self-perception, and external reality, as well as inadequacy and dullness of affection [1]. Schizophrenia affects more than 21 million people worldwide, and one in every two schizophrenia-afflicted subjects does not receive care for the disease [2], despite the fact that there are effective schizophrenia symptom-mitigation treatments and affected people can lead a productive life and be inserted into society [3].

Amongst the main schizophrenia manifestations the positive symptoms stand out, ordinarily termed psychotic symptoms, which include hallucinations and delusions, typifying the loss of the patient's contact with reality. Negative symptoms (mostly impairment of motivation, reduction of spontaneous speech, and social isolation) and cognitive impairment are also considered key features of this illness [4].

Hallucinations can be defined as the perception of an object, such as voice, noise, or image, without it being present, that is, without the respective sensorial stimulus [5]. They are experiences comparable to perception that occur without an external stimulus; it means that the patient experiences the behavior as not being under voluntary control [6]. In schizophrenia, hallucinations concerning visual, somatic, tactile, and olfactory modalities may take place, but the auditory ones are the most frequent indicators [7].

Over the past years, a greater access to technology has allowed the use of tools such as virtual reality (VR) in medicine. VR is an instrument capable of inserting the user into an interactive three-dimensional virtual world, using a computer that can generate images and a display that presents sensory information. Over the last few decades, the use of VR in psychotic disorders, such as schizophrenia, has been a promising candidate, for example, for a clearer understanding of symptoms, for training of instrumental and social skills, and for the treatment of the disease, as an adjuvant technique [8, 9]. With reference to the stigma, VR has been shown to be useful for diminishing negative stereotypes [10] and as a supporting device to increase empathy and positive impressions towards schizophrenic people [11] amongst other applications [12–24].

In addition to the severe symptoms of the disease, patients afflicted with schizophrenia face another key trouble: the stigma. Discrimination against these patients can be perceived when they try to make or maintain friendships, look for a job, or maintain intimate or sexual relations. Moreover, discrimination often comes from members of their own family [25]. In a study conducted in the United Kingdom, it was observed that more than 70% of the general public classified schizophrenia-afflicted people as dangerous or unpredictable [26].

One aspect to fight the stigma is intervention in specific groups of the population, such as police authorities and health providers. There are studies showing that training of police forces to recognize mental disorders could decrease the number of arrests and unnecessary force used against people with mental illnesses, as well as increase the adequate referral to psychiatric institutions [27].

Even though the effects of the stigma on the evolution of schizophrenia are still scarcely addressed by patients families and health practitioners, they have been observed in a number of studies. In recent times, it has been demonstrated, for instance, that stress instigated by stigma may be related to the transition to schizophrenia in young people at risk of psychosis [28]. Habitually, the patient feels downgraded. The disorder itself often leads to a condition of hypobulia and decreased motivation, which culminates with impaired physical conditioning. Environment-generated stimuli maybe cannot induce severe side effects, as there are no literary reports of serious symptoms generation (e.g., convulsive crisis) [29].

A number of approaches have been developed aiming at reducing the public stigma of mental illness and the damage it causes to the patient's life. For example, virtual environments to educate patients with schizophrenia of the hallucinations they suffer [30]. Amongst these approaches, social protest or activism, education of the public, and contact with mentally handicapped people stand out, while the latter two show positive results in the stigma reduction. Educational strategies comprise public messages, books, pamphlets, films, videos, web pages, virtual reality, and other audiovisual tools [31].

A healthy sample of the population submitted to the demonstration of the proposed environment can reduce interrelated obstacles, for example, to the delay in the beginning of the treatment and difficulties in interpersonal and professional relationships to have a proper approach to the conditions of people afflicted this serious mental illness.

The development of a VR-based tool capable of reducing the stigma related to schizophrenia is of utmost importance to treat this disease. To fill this gap, we propose a new augmented-reality-based tool capable of simulating the typical psychotic symptoms of schizophrenia and analyzing its effectiveness, the influence of the tool on stigmas reduction, emotional impact on users, and its validation by specialists and students of the medicine course by use and application of a questionnaire. The proposed system can interact, in real time, with a healthy sample of the population, medical students, simulating the psychotic symptoms typical of schizophrenia. The environment was developed with the support of a psychiatry specialist team.

Appraisal of the tool effectiveness is performed by Mental Health specialists through three questionnaires: schizophrenia-related stigma evaluation, environment simulation evaluation, and stigma evaluation after augmented-reality simulation. In the questionnaire assessing the schizophrenia-related stigma, volunteers' personal data, family experience in cases of schizophrenia-diagnosed persons, and a fictional history of a schizophrenia-afflicted individual were made. For the simulation evaluation, inquiries related to the performance of the AR environment were made. In the last questionnaire, the reassessment was made after an environment simulation of how the volunteer turned out to perceive the person from the fictional story about schizophrenia.

Environment-generated stimuli should not be able to trigger serious side effects, as there are no literature reports about serious symptoms induction (e.g., convulsive crises). Simulations can be applied in educational environments (school groups, colleges, and technical training) in professional settings such as hospitals and clinics, and in training and awareness-raising groups that may interact with psychosis-afflicted people, such as police officers, municipal guards, and family members of disease carriers.

By applying such virtual systems, connected with clinical purposes, they can play an important role in the healthcare area, as they are easily handled by the specialist as well as by the patient, being such a reassuring feature for the treatment continuousness [32]. For example, [9] presented a pilot study to investigate the feasibility and potential negative side effects of exposure to different virtual social risk environments in patients with first episode psychosis and in healthy controls.

## 2. Development of the Proposed Virtual Environment

Virtual and augmented reality are technological tools that use virtual elements that can be inserted in realistic scenarios, becoming an alternative and complementary approach to the treatments and diagnoses, being a very promising method to be adopted definitively in the health area [33].

Virtual reality is an advanced interface for computational applications in which users can navigate and interact with a three-dimensional computer-generated environment through multisensory devices. This technology establishes the relationship between the user and the created environment, allowing real-time integration with controlled virtual objects, and can simulate touch-screen monitors, mount-head goggles, mouse, and data glove and explore virtually freely, without real constraints like gravity, according to the immersion scenario and controls in which the user is interacting. In it, the individual has the sensation, in real time, of interacting with the virtual world manipulating objects around it [34].

Augmented reality is a type of advanced virtual reality interface that allows the user to experience sensations through stimuli to see, hear, feel, and interact with information and virtual elements inserted in the real environment [35].

In this work, the voices and figures were generated from the narrative of three schizophrenic patients treated at the NUEsq Schizophrenia outpatient clinic of Frota Pinto Mental Hospital in Fortaleza, Ceará. The data collection was qualitatively focused, based on the patients' verbal description of their psychotic experiences focusing on sensory perception alterations (auditory hallucinations and visual pseudohallucinations). The evaluation was led by a psychiatrist heading the clinical follow-up of the patients under discussion.

The tool consists of a software that reproduces some schizophrenia typical psychotic symptoms in an augmented-reality environment so that healthy people can undergo a simulation and thus provides a reality immersion for a patient carrying such ailment. In the environment, a 3-minute duration simulation was set, as it could become tiring over an extended period of time.

At the beginning, in the tool created in the Unity 3D program, a software that is used to create games and animations, an environment that could simulate what would be the reality was produced; namely, it was able to produce an immersion to the user as close to the reality as possible. To achieve this, a GameObject, named Cubo1 was designed, aimed at conveying with its texture a camera, so that when the operator used the environment he/she could visualize the real scenario in which he/she was inserted.

After the creation of the object that would use the camera to visualize the real-time environment, pictures that would make the process of simulation of the appearances of figures (common visual pseudohallucinations, referred by carriers) were inserted, with 2D sprites being used as scene GameObject, which could have their humanoid or geometric shapes a little rounded, and in them a blur effect was applied (blurred feature). The human-shaped figure has its animation modeled with fade-in and fade-out effects, causing it to appear or disappear at a particular point in time according to its program design, while its total four-second appearance is the most common. The geometric figure generally features the same outline when its animation is programmed similarly to the humanoid version, but in some cases the animation suffers the spawn effect (when the GameObject undergoes a transformation on the *x*-axis from one particular point to another), causing the GameObject to move from side to side, with a speed script being added. Additionally, the problem of the GameObject being “spawned” though never disappearing became evident, causing a future memory error. In consequence, a script was inserted so that it destroyed itself at a given time.

In the facial detection phase, a Unity plugin called OpenCV for Unity, which is a plugin that makes Unity recognize OpenCV scripts used in facial detection, was used. In the development of the facial modification a sprite (2D animated image) of a blurred spot was used to cause a somber effect in the interviewees' gaze. The effect of the facial modification lasted about 1 second, running twice during the three minutes.

The voices heard in the simulation via headphones were digitally recorded by two actors from a script prepared by the psychiatrist who piloted the interview with the volunteer patients. The audio plays a generic content, usually heard by patients with auditory hallucinations: whispers, threatening and commanding speech, laughter, and offending content phrases. Two voices were chosen, one male and one female, based on recurrent schizophrenia hallucinatory phenomena involving voices of different characteristics that often talk to each other. The choice of actors and nonrobotic voices was aimed at promoting authenticity and naturalness, which are distinctive of true hallucinations.

All students gave their informed consent to the procedure, which was approved by the Ethics Committee of Escola de Saúde Pública do Ceará, Ceará, Brazil (permit 1.569.510, June 1, 2016).

### 2.1. System Architecture

**Figure 1:**
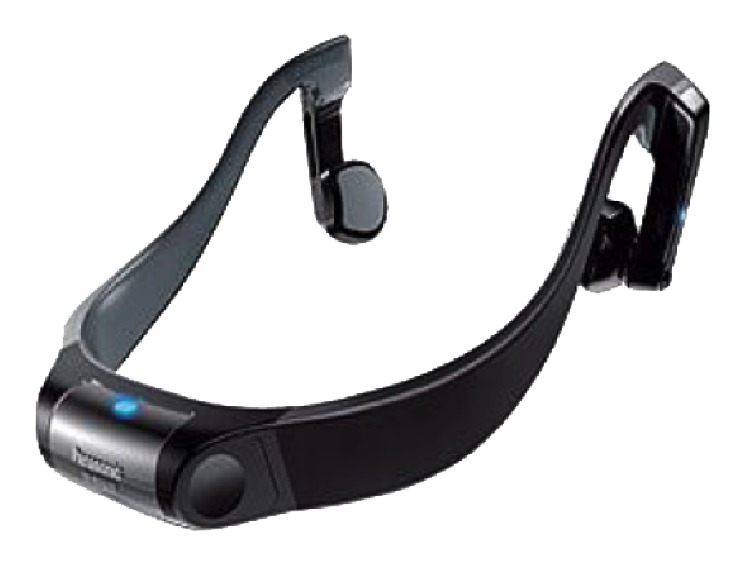
Panasonic phone RP-BTGS10.

**Figure 2:**
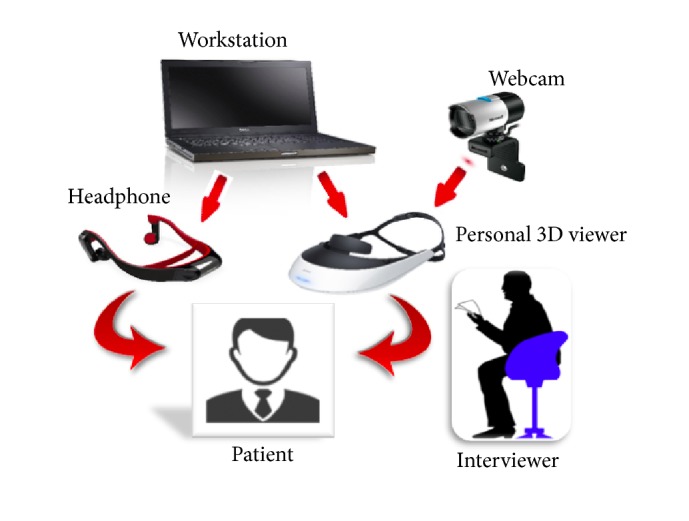
Device integration.

The implementation of the proposed system is based on the use of Unity as an engine of the augmented-reality environment, being used as 2D sprites scene objects produced in an image editing software. The HMZ-T2, Sony Glasses, responsible for transmitting the image processor, coupled with a LifeCam Studio Microsoft is placed in the user's head with a Panasonic RP-BTGS10 phone shown in Figure 1, which uses the bone to convey sound vibrations to the nervous system, transporting the sound of the environment and not interfering in the interviewer's voice.  Figure 2 shows the integration of the devices in the system.

### 2.2. Environment Integration with the Equipment

As the purpose of this paper was to convey the psychotic symptoms to a healthy sample of the population, it demanded a good interaction of the tool with the equipment employed. The tool consists of software that can be installed on any computer that has Windows operating system with versions from Win 7. It already has a simple interface that was created by the graphic engine Unity 3D itself, being easily accessible to any user.

### 2.3. Modeling and Animation

#### 2.3.1. Modeling and Audio Animation

Sound hallucinations were created by means of sound editing software and recorded with a microphone that was emitted by a headset. In creating the audio hallucinatory phrases noises, whispers and provocations, like “you aren't worth a thing” type were implemented. Some order phrases with “he will kill you” or “get out of here” were used in the environment audio. While developing the audio some pauses were made. In order to give more immersion as if a person were whispering in the ear, the procedure was applied in each and every phrase or any sound emitted by the environment, by using a sound channel coming out separately on each side of the headset. In the environment, the user of the glasses should listen to the voice of the interviewer who would make questions to the user while he/she listened to the sound effects.

#### 2.3.2. Modeling and Figure Animation

**Figure 3:**
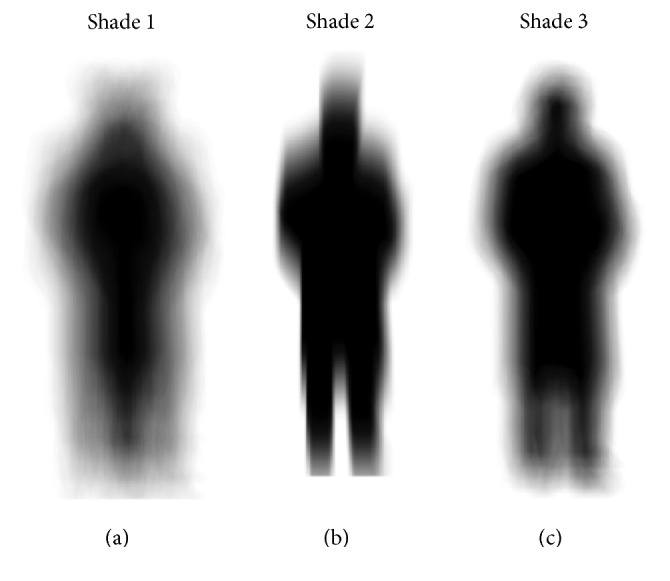
Models of figures with man silhouette: (a) more blur; (b) less blur; (c) middle blur.

**Figure 4:**
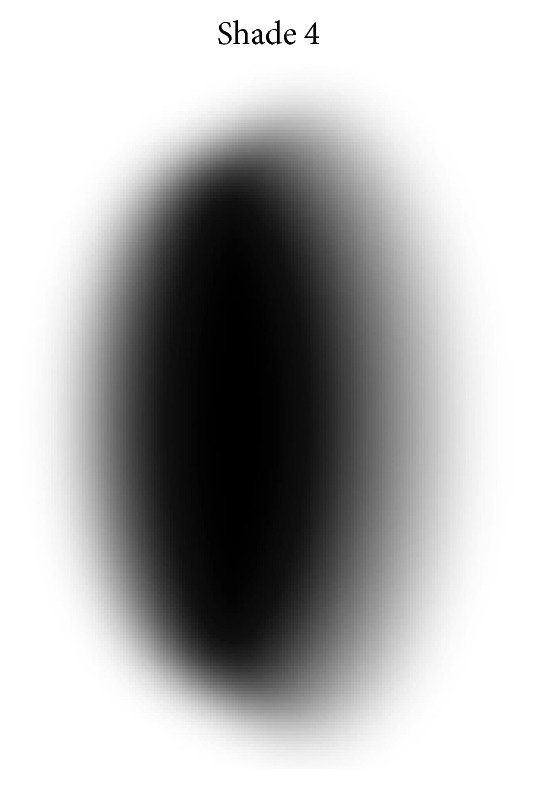
Flat circumference figure model.

The figure, modeled on an imaging software and animated in Unity, plays a major role in the augmented-reality environment, because it simulates one of the typical schizophrenia psychotic symptoms. This is the case when a patient has hallucinations in his/her vision that cause a perception of something nonexistent, but that is a subjective impression that it is something real without external stimulus. Consistent with reports from patients the figure may have a human appearance or a blur. To develop the figure images of a man silhouette and a flattened circumference were used, and then the blur effect was applied so that the image provided an impression of an image with twisted visual focus.  Figure 3 shows model types made of figures for the environment with man silhouette, where there are more blur in Figure 3(a), less blur in Figure 3(b), and medium blur Figure 3(c).  Figure 4 illustrates the model of a circumference flattened by the sides. The animations were constructed so that there was some relation with what was being heard with the audio mentioned. The figure underwent a transformation of position on the *x*-axis corresponding to the camera and appeared in parts demarcated by the studies. The other figures, which are the man silhouette, appeared in the extreme part of the camera view, while the fade effect was applied by varying the fade-in and fade-out time according to the sound effects.

#### 2.3.3. Modeling and Animation of Facial Transformation Effect

In facial modification, the OpenCV plugin for Unity available in the Unity Asset Store was used. Plugins function to include the use of OpenCV in Unity. As this package is an OpenCV Java clone, we can use the same API with OpenCV Java 3.1.0. It works with Unity free and pro with iOS, Android, Windows Store Apps 8.1 support, Windows Phone 8.1, Windows 10 UWP support (beta), Support Win, Mac, and Linux Standalone Support for Editor viewing. Its image processing is in real time using the Drive WebCamTexture capabilities (real-time face detection works flawlessly on iPhone 5). Version 1.9.0 of the plugin was used. With this plugin, we can proceed with the facial recognition and thus apply deformation and facial modification effects. In this work, to perform the facial modification, a dark circumference, see Figure 4, was placed over the eyes, accompanying the movements of the user's face through a 2D sprite provided by Unity Technologies for use in prototyping of games.

The process is aimed at simulating psychopathological changes experienced by the schizophrenia-afflicted individuals during their psychotic symptoms. Some patients report illusions involving the face of interlocutors, who may appear to patients as gloomy or even monstrous. As such, the simulated effect causes discomfort and estrangement feelings, rendering the experience more threatening.

## 3. Results

As a result of this work, a simulator was developed in an augmented-reality environment called “Schizophrenia Simulator” originally designed as the proposal in this work, so that the environment could be evaluated as to the possibility of being used as a tool to help reducing the stigma with reference to schizophrenia.

In this work, the analysis was made through observations and analysis of the questionnaire based on multiple comparison to evaluate the significance of the changes in the observed parameters.

### 3.1. Screenshots of the Developed Environment and Testing Used

**Figure 5:**
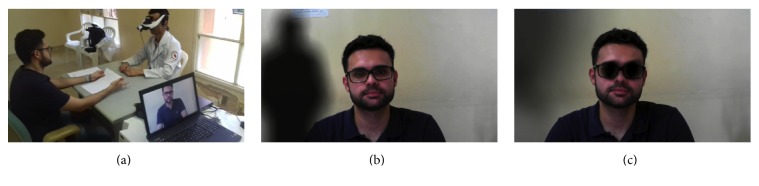
(a) Environment simulation with the medical student; (b) visualization in the simulation; (c) visualization of the facial modification in the simulation.

Figure 5(a) shows the use of an environment simulation tool by a medical student, analyzing his behavior during his interaction with the augmented-reality environment through virtual reality glasses, as well as the usability of the tool for possible adjustments for difficulties presented by users. From this test, it was acknowledged that initially the user had difficulties adjusting the glasses on his head, because in some cases the device did not fit properly because of its size. The infrastructure used for this test was a doctor's office of Frota Pinto Mental Hospital. A medical consultation was performed using a simulator, in which the volunteer (medical student) would answer some questions in a personal, professional, and logical reasoning scheme at a time when the schizophrenia psychotic-symptom simulating environment was running.  Figure 5(b) shows how the medical student (patient) sees the visual-effect simulating figures of a schizophrenia psychotic symptom.  Figure 5(c) shows the facial modification effect applied in the environment visualized by the medical student (patient) in real time.

### 3.2. Evaluation of the Virtual Environment

In order to validate the augmented-reality environment, medical students at Mental Hospital Frota Pinto were invited to take part in a simulation of augmented-reality environment using virtual reality glasses. They had the opportunity to use it to explore their possibilities and to analyze the feasibility of this augmented-reality environment as an alternative to help in decreasing the schizophrenia-related stigma. Twenty-one students from three universities in the state of Ceará were present for the environment trial. After using the augmented-reality environment, the medical students were submitted to three questionnaires: evaluation of the schizophrenia-related stigma, evaluation of the environment simulation, and evaluation of stigma after an augmented-reality simulation. According to Pfleeger (1994) and Wohlin et al. (2000), a questionnaire should be applied to analyze quantitative data before and/or after application of an approach.

The aim of the questionnaires was to be used as a prestudy in order to certify that important issues related to the study were anticipated, as well as to depict expectations, perceptions, and opportunities related to the possibility of the actual use of an augmented-reality environment in users as a complementary tool for understanding, stigma reduction, and prejudice. The questionnaire to assess the schizophrenia-related stigma consisted of 9 questions; the last one was related to José's story, a fictitious schizophrenic patient, in his first contact with the user, and the other 8 questions have a personal nature and asked whether there are cases of family members diagnosed with schizophrenia. The other questionnaires (Table 1 to Table 4) correspond to the evaluation of the proposed virtual environment on stigma reduction related to schizophrenia. In the last questionnaire evaluating the stigma after an augmented-reality simulation, questions related to José's story with a postsimulation feature were made.

Analyzing the results of the questionnaires application, see Table 1, it was found that in the questionnaire related to the environment simulation evaluation, on a 1–5 scale, of not at all to extremely, 42.85% of medical students chose the value 5, 42.85% of the students considered 4, and 14.30% chose 3, regarding the realism of the proposed environment in the execution of its sound effects; 23.80% of the medical students considered on a 1–5 scale, of not at all to extremely, value 5 and 47.60% considered 4, 14.20% of the medical students chose 3, and the same value chose 2, regarding the realism of the proposed environment in the execution of its visual effects. With regard to the augmented-reality environment proposed, there is the sensation of reality immersion (feeling of belonging to reality): 9.50% of the medical students considered on a 1–5 scale, of not at all to extremely, the value 5, while 57.15% considered 4 and 28.60% of the medical students chose 3 and 4.75% chose 2. On the other hand, 80.95% and 19.05% of the medical students, on a 1–5 scale, of not at all to extremely, chose 5 and 4, respectively, if the simulation was educational concerning the schizophrenia symptoms. Regarding whether the simulation should make users more empathetic towards schizophrenia-afflicted individuals, on a 1–5 scale, of not at all to extremely, 71.40% considered the value 5, 19.05% considered 4, 4.75% indicated 3, and the same value, 4.75%, chose 2. Table 1 shows the results quantitatively found with the objective questions of the questionnaire.

Questions on the environment simulation evaluation questionnaire related to the symptoms experienced during or shortly after the simulation reported that 19.05% of the medical students had a general malady (a general sensation of discomfort and uneasiness), fatigue, and “heavy headache” (due to the equipment weight); 4.76% said that they had problems with headache, dizziness with open eyes, and abdominal discomfort (stomachache). On the other hand, 38.10% reported they had a tired eye and blurred vision. It was found that 71.40% of the medical students had difficulty maintaining focus and concentration (seeing clearly and getting confused with the voices of the environment). 9.52% of the users reported sweating problems (perspiration or sweating) and nausea (an uncomfortable stomach feeling) and 14.30% reported dizziness with open eyes.  Table 2 shows the analysis of the medical students' answers about the symptoms felt during or shortly after the simulation.

Regarding the subjective questions, the medical students emphasize that the proposed environment stands out.

*Positive Points*. Positive points are as follows: being educational for health professionals and others, simulation in a suitable place rendering realistic effects, experience in understanding a psychotic patient's mind, reducing stigma and prejudice, showing the reality experienced by a schizophrenic, understanding the disease symptoms, interactivity being more attractive than the conventional information practice, and empathy with the schizophrenia symptoms.

*Negative Points*. Negative points were as follows: difficulty in putting glasses on the head, discomfort using the device, simulating in a more restless setting, difficulties in viewing for glasses users, voice-audio synchronization improvement, and possibility of causing uneasiness for some individuals.

*Possibilities for Improvement*. Possibilities for improvement include improving the external environment lighting, use of VR glasses with a better and more comfortable fitting and better closing of vision, adding real people in simulations, and figure-audio synchronization improvement.

In order to evaluate the impacts on the schizophrenia-related stigma, a questionnaire that evaluated questions about a fictional patient called “José” was applied, and these same questions were applied after using an augmented-reality environment, assessing the result changes. Appendix 2 of the questionnaire tells José's story. Questions and inquiries in the questionnaire comprised the following: “Would I feel sorry for José?,” “How dangerous do you think José is?,” “How scared would you feel with José?,” “I think José is guilty of his present condition,” “I think it would be better for José's community that he be admitted to a psychiatric hospital,” “How angry would you feel about José?,” “How likely is it that you will help José?,” “I would try to stay away from José,” and “Do you think José should be forced to take medical treatment, even against his will?” on a 1–9 scale, in which 1 corresponds to “no or nothing” and 9 corresponds to “very or completely.”

During the results analysis of the application of the questionnaire related to the evaluation of the medical students' responses (Table 3) on the schizophrenia-related stigma, on a 1–9 scale of no/nothing and very/completely, 28.57% of the medical students gave value of 9; 23.80% considered 8; 14.28% chose 7; 6. 9.52% indicated value 5; 4.6% picked 4 and 3 for item “I would feel sorry for José,” 4.76% of the medical students on a 1–9 scale of no/nothing and very/completely considered value 8, while 9.52% indicated 7; 14.20% recorded 6; 23.80% chose 5; 9.52% took 4 and 19.04% selected items 3 and 2 on the scale for item “How dangerous do you think José is?” Concerning the “How scared would you feel with José?” item, on a 1–9 scale of no/nothing and very/completely, 9.52% of the medical students scored values of 8, 6, 5, and 4; 4.76% indicated 7; 23.80% picked value 3; 14.28% chose value 2; and 19.04% scored 1. 9.52% of the medical students on a 1–9 scale of no/nothing and very/completely, item “I think José is guilty of his present condition” chose value of 3, and 90.47% picked value 1. Regarding item “I think it would be better for José's community that he be admitted to a psychiatric hospital” on a 1–9 scale of no/nothing and very/completely, 4.76% of the medical students pointed out values of 9, 7, 6, and 3. 9.52%, 19.04%, and 53.38% chose 5, 2, and 1, respectively. 4.76% of the medical students on a 1–9 scale of no/nothing and very/completely chose values 4 and 3, 28.57% selected 2, and 61.90% marked value 1 concerning item “How angry would you feel about José?” Values 9 and 5 were marked by 9.52% each. 28.57% selected value 8. 33.33% chose 7, 14.28% considered 6 and 4.76% scored 4 on a 1–9 scale of no/nothing and very/completely of item “How likely is it that you will help José?” In regard to “I would try to stay away from José,” on a 1–9 scale of no/nothing and very/completely, 14.20% chose 7, while 19.04% selected 6 and 2; 4.76% considered 4. 9.52% chose 3 and 33.33% picked 1. On a 1–9 scale of no/nothing and very/completely relative to item “Do you think José should be forced to take medical treatment, even against his will?” 14.28% of the medical students considered values 9, 8, and 1, 4.76% selected 7, 6, and 2, 23.80% marked 5, and 19.04% pointed out 4. Table 3 shows the analysis of the medical students' responses about the schizophrenia-related stigma evaluation.

During the results analysis of the application of the questionnaire related to the evaluation of the medical students' answers (Table 4) on the evaluation of stigma after augmented-reality simulation, on a 1–9 scale of no/nothing and very/completely, 66.66% of the medical students marked value 9, 4.76% of the students considered 8, and 9.52% picked values 7, 6, and 5 concerning item “Would I feel sorry for José?” On a 1–9 scale of no/nothing and very/completely of item “How dangerous do you think José is?”, 19.04% of the medical students considered values 8 and 7, 9.52% chose values 6, 5, and 4, 14.28% marked values 3 and 2, and 4.76% scored 1. Concerning item “How scared would you feel with José?,” on a 1–9 scale of no/nothing and very/completely, 9.52% scored 9, 5, 4, and 1, while 14.28% scored values 7 and 2; 4.76% of the medical students considered 8 and 6, and 23.80% selected value 3. On the 1–9 scale of no/nothing and very/completely of item “I think José is guilty for his present condition.” 90.47% picked value 1, and 4.76% of the medical students considered values 5 and 2. In item “I think it would be better for José's community that he be admitted to a psychiatric hospital,” on a 1–9 scale of no/nothing and very/completely, 19.04% of the medical students scored value 9. 9.52% chose value 6, 14.28% considered each 5 and 2, 4.76% scored 4 and 3, and 33.33% selected value 1. 4.46% of the medical students on a 1–9 scale of no/nothing and very/completely, for item “How angry would you feel about José?,” chose values 4 and 2, 76.19% chose value 1, and 14.28% considered value 3. For item “How likely is it that you will help José?,” on a 1–9 scale of no/nothing and very/completely, 9.52% of the medical students scored 8 and 5, 14.28% took 7 and 6, 52.38% scored value 9. 9.46% of the medical students considered, on a 1–9 scale of no/nothing and very/completely, values 8, 6, and 3, while 14.28% chose 5 and 4; 19.04% of the medical students chose 2; and 38.09% scored 1, for item “I would try to stay away from José.” On a 1–9 scale of no/nothing and very/completely, 28.57% of the medical students considered value 9, 14.28% of the students chose 8 and 2, 9.52% chose 7, 23.80% scored value 5, and 4.76% chose values 3 and 1 for item “Do you think José should be forced to take medical treatment, even against his will?” Table 4 shows the analysis of the medical students' responses on the evaluation of stigma after the augmented-reality simulation.

After the simulation, as stated by the questionnaires, there was a trend to help the fictitious case patient. It is important to note that there was also an increase in the trend to hospitalize the patient and to treat him/her involuntarily.

These data indicate a change of posture after the simulation: while there was an increase in empathy, there was an apparent greater impression that the presented picture is serious. There was also an ascending trend after the simulation of considering José dangerous.

The results demonstrated an increase in the mean stigma score from 32.05 to 35.38 (*p* = 0.004), with statistical significance in pity, fear, and segregation. On the other hand, there was an increase in the average score referring to the probability of giving help, from 7.00 to 7.81 (*p* = 0.034). The tool had good acceptance, demonstrated by the average scores, for use as a teaching method (4.8), ability to increase the understanding of people with schizophrenia (4.57), realism of sound effects (4.29), realism of visual effects (3.81), and sensation of immersion in reality (3.71), on a scale from 1 (not at all) to 5 (extremely). In the analysis of the emotional impact of the tool on individuals submitted to the proposed environment, there was always some sort of symptom, and the most common one was the difficulty of maintaining focus and concentration.

Although this was an isolated intervention, there was an increase in the mean total stigma score; moreover, the simulation of psychotic symptoms through the augmented reality also resulted in increased scores of the possibility of helping a patient with psychosis. In general, the proposed system obtained a good overall evaluation. Notwithstanding these positive points, further improvements in the technique will be needed in order to decrease discomfort and improve concerns related to size and portability, so that the tool can have a large-scale usage. However, in its present form, the new tool can be used as an educational resource so that people can become more familiar with the symptoms of schizophrenia, and in doing so reducing its stigma and enhancing empathy for the patients.

The design of this study has limitations related to the long-term effect of VR in the change of attitude towards the bearer of schizophrenia. In cross-sectional analysis it is impossible to predict whether the experiment is capable of causing lasting stigma reduction.

## 4. Conclusion

This work presented the development of an augmented-reality environment simulating the schizophrenia psychotic symptoms as an alternative tool to help reducing the schizophrenia-related stigma so that there is no delay at the beginning of treatment. We specifically addressed the use of an augmented-reality environment through Sony HMZ-T2 glasses, which proved useful for the simulation of visual and auditory effects, managing to transmit the visual effects well with few exceptions, such as people wearing glasses, who could not see appropriately. The Panasonic RP-BTGS10 headset, which uses bone tissue to transfer sound vibrations to the nervous system, is promising, lightweight, and affordable.

According to the results, the medical students and specialist/psychiatrist included the use of the augmented-reality environment as an educational resource for health professionals as well as for family members and society in search of reducing the disease-related stigma. In the analysis of the emotional impact of the tool on individuals submitted to the proposed environment, it shall be noticed that there was always some sort of symptom, while the difficulty of maintaining focus and concentration was the most common one.

The tool developed in this work showed very promising results, and can be applied in educational environments (groups of schools, colleges, and technical training), in hospitals and clinics, and in the training and awareness-raising of groups that may come into contact with psychosis-afflicted patients, such as police officers, municipal guards, and family members, amongst many other applications. It will be necessary to further improve the technique in order to decrease discomfort and improve concerns related to size and portability, so that it can have a large-scale application. In general, the new tool can be used as an educational resource so that people become more familiar with the schizophrenia symptoms, and in so doing reducing its stigma and enhancing empathy for the patients.

Longitudinal evaluation studies with one or multiple exposures to the RV tool can demonstrate if the effects of the experiment are lasting over time, as well as determining the need to repeat the experiment to consolidate the expected effect. Future studies may also indicate whether the effect of immersion experience can be reinforced by supplementary psychoeducation measures such as short lectures on schizophrenia, for example.

## Figures and Tables

**Table 1:** Questions and analysis of the medical students' responses on the proposed augmented-reality environment.

Objective questions made	Extremely [%]			Little [%]	
	5	4	3	2	1
(1) Does the proposed environment ensure realism in the execution of its sound effects?	42.85	42.85	14.30	—	—
(2) Does the proposed environment ensure realism in the execution of its visual effects?	23.80	47.60	14.20	14.20	—
(3) Does the proposed environment create a sensation of immersion in reality (feeling of belonging to reality)?	9.50	57.15	28.60	4.75	—
(4) Was the simulation educational about schizophrenia symptoms?	80.95	19.05	—	—	—
(5) Should simulation make users more empathetic towards people with schizophrenia?	71.40	19.05	4.75	4.75	—

**Table 2:** Analysis of medical students' answers about the symptoms felt during or shortly after the simulation.

Questions related to symptoms	Reports [%]
(1) General malady	19.05
(2) Weariness	19.05
(3) Headache	4.76
(4) Eyestrain	38.10
(5) Difficulty to maintain focus	71.40
(6) Increased salivation	—
(7) Sweating	9.52
(8) Nausea	9.52
(9) Difficulty to concentrate	71.40
(10) “Heavy head”	19.05
(11) Blurry vision	38.10
(12) Dizziness with open eyes	14.30
(13) Dizziness with closed eyes	4.76
(14) Vertigo	—
(15) Abdominal discomfort	4.76
(16) Belch	—

**Table 3:** Questions and analysis of medical students' responses about the schizophrenia-related stigma evaluation.

Question/statement	No/nothing [%]					Very/completely [%]			
	1	2	3	4	5	6	7	8	9
I would feel sorry for José.	—	—	4.76	4.76	9.52	14.28	14.28	23.80	28.57
How dangerous do you think José is?	—	19.04	19.04	9.52	23.80	4.28	9.52	4.76	—
How scared would you feel with José?	19.04	14.28	23.80	9.52	9.52	9.52	4.76	9.52	—
I think José is guilty of his present condition.	90.47	—	9.52	—	—	—	—	—	—
I think it would be better for José's community that he be admitted to a psychiatric hospital.	52.38	19.04	4.76	—	9.52	4.76	4.76		4.76
How angry would you feel about José?	61.90	28.57	4.76	4.76	—	—	—	—	—
How likely is it that you will help José?	—	—	—	4.76	9.52	14.28	33.33	28.57	9.52
I would try to stay away from José.	33.33	19.04	9.52	4.76	—	19.04	14.28	—	—
Do you think José should be forced to take medical treatment, even against his will?	14.28	4.76	—	19.04	23.80	4.76	4.76	14.28	14.28

**Table 4:** Questions and analysis of medical students' answers on the evaluation of stigma after augmented-reality simulation.

Question/statement	No/nothing [%]					Very/completely [%]			
	1	2	3	4	5	6	7	8	9
I would feel sorry for José.	—	—	—	—	9.52	9.52	9.52	4.76	66.66
How dangerous do you think José is?	4.76	14.28	14.28	9.52	9.52	9.52	19.04	19.04	—
How scared would you feel with José?	9.52	14.28	23.80	9.52	9.52	4.76	14.28	4.76	9.52
I think José is guilty of his present condition.	90.47	4.76	—	—	4.76	—	—	—	—
I think it would be better for José's community that he be admitted to a psychiatric hospital.	33.33	14.28	4.76	4.76	14.28	9.52	—	—	19.04
How angry would you feel about José?	76.19	4.76	14.28	4.76	—	—	—	—	—
How likely is it that you will help José?	—	—	—	—	9.52	14.28	14.28	9.52	52.38
I would try to stay away from José.	38.09	19.04	4.76	14.28	14.28	4.76	—	4.76	—
Do you think José should be forced to take medical treatment, even against his will?	4.76	14.28	4.76	—	23.80	—	9.52	14.28	28.57
